# Myocardial Scintigraphy in the Evaluation of Cardiac Events in Patients without Typical Symptoms

**DOI:** 10.5935/abc.20150074

**Published:** 2015-08

**Authors:** Paola Emanuela Poggio Smanio, Juliana Horie Silva, João Vitor Holtz, Leandro Ueda, Marilia Abreu, Carlindo Marques, Leonardo Machado

**Affiliations:** Instituto Dante Pazzanese de Cardiologia, São Paulo, SP – Brazil Mailing

**Keywords:** Myocardial Scintigraphy, Ischemia, Atypical Symptoms, Hard events

## Abstract

**Background:**

Cardiovascular disease is a leading cause of death in the world and in Brazil. Myocardial scintigraphy is an important noninvasive method for detecting ischemia in symptomatic patients, but its use in asymptomatic ones or those with atypical symptoms is yet to be defined.

**Objective:**

To verify the presence of major cardiac events in asymptomatic patients or those with atypical symptoms (atypical chest pain or dyspnea) that underwent myocardial scintigraphy (MS), over a period of 8 years. Secondary objectives were to identify cardiac risk factors associated with myocardial scintigraphy abnormalities and possible predictors for major cardiac events in this group.

**Methods:**

This was a retrospective, observational study using the medical records of 892 patients that underwent myocardial scintigraphy between 2005 and 2011 and who were followed until 2013 for assessment of major cardiac events and risk factors associated with myocardial scintigraphy abnormalities. Statistical analysis was performed by Fisher’s exact test, logistic regression and Kaplan-Meyer survival curves, with statistical significance being set at p ≤ 0.05.

**Results:**

Of the total sample, 52.1% were men, 86.9% were hypertensive, 72.4% had hyperlipidemia, 33.6% were diabetic, and 12.2% were smokers; 44.5% had known coronary artery disease; and 70% had high Framingham score, 21.8% had moderate and 8% had low risk. Of the myocardial scintigraphies, 58.6% were normal, 26.1% suggestive of fibrosis and 15.3% suggestive of ischemia. At evolution, 13 patients (1.5%) had non-fatal myocardial infarction and six individuals (0.7%) died. The group with normal myocardial scintigraphy showed longer period of time free of major cardiac events, non-fatal myocardial infarction (p = 0.036) and death. Fibrosis in the myocardial scintigraphy determined a 2.4-fold increased risk of non-fatal myocardial infarction and five-fold higher risk of death (odds ratio: 2.4 and 5.7, respectively; p = 0.043).

**Conclusion:**

The occurrence of major cardiac events in 8 years was small. Patients with fibrosis at MS had more major events, whereas patients with normal MS result had fewer major cardiac events, with higher survival.

## Introduction

Cardiovascular diseases are the leading cause of death worldwide, with ischemic heart disease and cerebrovascular accident (CVA) being the most frequent^1^.

The incidence of Coronary Artery Disease (CAD) is increasing worldwide, being the second leading cause of death in Brazil, with a mean of 80,000 deaths per year. Its prevalence in the adult population is estimated at 5-8%^2^. According to the Department Informatics of the Brazilian Unified Health System (DATASUS), in 2010, ischemic heart disease accounted for 210,046 hospital admissions in Brazil and 29% of deaths (99,408 deaths or 55.11 deaths/100,000 inhabitants)^3,4^, corresponding to a burden of R$ $ 1.9 billion, or 19% of the total cost with hospitalizations^5^.

Due to the epidemiological importance of CAD, appropriate risk stratification strategies are needed to establish better cost-effectiveness and safety of preventive treatments, as well as request for additional tests.

The clinical manifestation of coronary heart disease is the result of the imbalance between myocardial oxygen supply and consumption. The subjective description of angina may make symptom interpretation difficult and, therefore, the clinical diagnosis. Thus, objective ischemia tests can confirm the diagnostic hypothesis and assess CAD severity^6^.

Myocardial scintigraphy (MS) is a cornerstone in the evaluation of patients with suspected CAD due to its high diagnostic accuracy, as well as being able to define the extent, severity and location of myocardial perfusion abnormalities, greatly assisting in clinical management^7^.

The technique uses electromagnetic gamma radiation to obtain images. Radioactive isotopes are injected into the patient and, due to affinity for the myocardium, they bring perfusion and/or metabolic information. After the radiotracer injection, it is possible to indirectly assess blood flow and myocardial flow reserve in a non-invasive manner^8,9^.

Currently, there is strong evidence for using MS in the diagnosis, follow-up, risk stratification and prognosis of symptomatic patients with known or suspected CAD. However, MS indication in asymptomatic patients or patients with atypical symptoms, even with known CAD, is yet to be defined, since in addition to the fact that benefits in this population are not fully established, the examination involves the inherent risks of physical or pharmacological stress, as well as exposure to ionizing radiation^10,11^.

The objective of this study was to identify, in asymptomatic patients or patients with atypical symptoms submitted to MS, the occurrence of events such as death and acute myocardial infarction (AMI), occurring in up to 8 years. Secondary objectives were to define the time free of events such as death and AMI after normal MS in asymptomatic patients or patients with atypical symptoms; to identify risk factors associated with alterations in myocardial perfusion scintigraphy; to identify risk factors that are independent predictors of death and nonfatal AMI in this group of patients.

## Methods

Retrospective, observational study carried out by the data analysis of medical records in a group of asymptomatic patients with cardiovascular symptoms or those considered to be atypical, with previously known CAD or not.

All patients underwent MS at Instituto Dante Pazzanese de Cardiologia from December 2005 to June 2011. Patients were followed for the period from the date of the examination until July 2013, to verify the occurrence of nonfatal myocardial infarction or death. All patients were analyzed for the presence of systemic arterial hypertension (SAH), dyslipidemia, smoking status, Occlusive Peripheral Arterial Disease (OPAD) and / or carotid disease, diabetes mellitus, chronic renal failure (CRF), ischemic cerebrovascular accident (iCVA), previously known CAD, Left Ventricular Ejection Fraction (LVEF) < 50%, family history of coronary heart disease, type of stress used at the MS (exercise stress or pharmacological stress test with dipyridamole), and presence of alteration suggestive of ischemia in the exercise test performed prior to the MS.

Patients were also classified as having high, intermediate and low cardiovascular risk, according to the Framingham score. A patient was considered hypertensive if he/she required the use of one or more antihypertensive drugs and through the criteria used in the VI Brazilian Guidelines on Hypertension of the Brazilian Society of Cardiology^11^; and was considered dyslipidemic according to the criteria of the IV Brazilian Guidelines on Dyslipidemia and Atherosclerosis Prevention of the Department of Atherosclerosis of the Brazilian Society of Cardiology^12^. A patient was considered diabetic when he or she required the use of one or more oral hypoglycemic agents and/or insulin and no patients with metabolic syndrome were included. The presence of CRF was defined if the patient had creatinine clearance < 90 mL/min; LVEF was determined by Doppler echocardiogram performed at most three months before the MS with no procedures between the methods.

MS was performed using^99m^ Tc-sestamibi as the radiotracer and according to the standard protocol of 1 or 2 days, with the stress test (exercise or pharmacological test) being the basal stage, being performed on the same day or on subsequent days.

Inclusion criteria were patients submitted to MS that did not show any cardiac symptom from the date of the examination request by the requesting clinician to the date when the MS was performed; and those with symptoms considered atypical by the clinician that requested the examination, as they did not meet the classic characteristics described, such as angina (retrosternal pain, triggered by exertional or emotional stress, with relief at rest or nitrate) or that had symptoms suggestive of ischemic equivalent (dyspnea). Most of the patients had other cardiac symptoms that were poorly characterized and uncharacteristic for ischemic heart disease.

Exclusion criteria were patients with chest pain suggestive of ischemic heart disease, dyspnea or symptoms suggestive of ischemic equivalent, or electrocardiogram suggestive of ischemia from the date of the MS request until its performance; and patients with incomplete data during the review of medical records.

The MS were analyzed by two specialists in nuclear medicine and a third expert was called in to analyze the images, when there was disagreement. The images were analyzed qualitatively only by the presence or absence of low radiotracer uptake in the myocardium in the 17 analyzed segments.

The equipment used for image acquisition was the Millennium VG gamma camera (GE Medical Systems, Milwaukee, United States), with two scintillation detectors, angled at 90°, with parallel hole collimators, high resolution and low energy. Information acquired was processed in a Xeleris workstation.

MS was performed associated with physical exertion with exercise testing or pharmacological stimulation through dipyridamole infusion, according to clinical indication. Bruce and modified Bruce protocols were used at the exercise testing, with anti-ischemic medications being withdrawn according to standardized recommendations from the nuclear medicine department.

The clinicians of the institution were aware of the drug withdrawal for the test and, when they did not wish the medication to be withdrawn they requested that the evaluation should be performed while on medication, in the medical request form. The following were considered as ischemic myocardial response criteria to the physical stress test, as standardized in the literature: the presence of ST‑segment depression ≥ 1.5 mm during or after exercise, when compared to baseline, with slow ascending morphologies (analyzed at point Y), horizontal (analyzed at point J) or descending (analyzed at point J), or the presence of clinical signs/symptoms suggestive of ischemia, according to the known classical and standardized criteria^13^.

For the pharmacological test, dipyridamole was infused at a dose of 0.56 mg /kg/ min during a total time of 4 minutes. A dose of 20 mCi or 740 MBq of 99mTc-MIBI was administered in the second minute after the dipyridamole infusion was finished, considered as the moment of maximum hyperemia. As interpretation criteria of electrocardiographic response to dipyridamole, used for the characterization of the ischemic response, the presence of horizontal depression, slowly ascending or descending ST segment ≥ 1.0 mm (or intensification of depression = 1.0 mm) was considered suggestive of ischemia, measured at the J point in the horizontal and descending morphologies, and at the Y point, in the slow ascending morphology. Typical chest pain and/or other clinical manifestations suggestive of coronary heart disease were also considered suggestive of ischemia, according to known classical and standardized criteria^13^.

The present study only analyzed the presence or absence of alterations in the functional tests that were suggestive of ischemia and prognostic scores were not calculated.

The images were processed using the dedicated software QGS, also known as Cedars-Sinai software, obtaining tomographic cuts in the vertical plane, according to the smallest cardiac axis, in the vertical plane, according to the greatest axis and in the horizontal plane, according to the greatest axis. The cuts of the two phases were paired to allow a cut-to-cut comparison of the radioactive concentration at stress and at baseline.

Image processing synchronized with the ECG provided the reproduction of cardiac wall contractions, in addition to indices of systolic and diastolic volumes and LVEF; it was possible to visualize the heart dynamics in both views, as well as of a three-dimensional representation of the left ventricle.

According to the department routine, the criteria for the analysis of myocardial perfusion images were visual and qualitative evaluations of the radiotracer concentration in the different myocardial segments (17 segments), comparing cut‑by-cut the images of the stress phase with the corresponding baseline ones. The following factors were considered in the qualitative analysis of myocardial perfusion: homogeneity or heterogeneity of the radiotracer concentration in myocardial segments; extension of radiotracer concentration defects in the myocardial segments; and intensity of the relative low uptake of the radiotracer in the myocardial segments. However, for this study, we took into account only the presence or absence of perfusion abnormalities.

MS was considered normal if the radiotracer concentration was homogeneous in both phases (basal and stress); suggestive of ischemia, if the low uptake was reversible after stress; suggestive of fibrosis, if the low uptake was fixed after the stress phase in relation to baseline; and suggestive of ischemia and fibrosis, if there was fixed and reversible low uptake of the radiotracer in one or more myocardial segments at the baseline and stress phases.

Statistical analysis was performed using the Statistical Package for Social Sciences (SPSS), and Pearson’s chi-square test and Fisher’s test were used, with statistical significance being set at p < 0.05. Moreover, logistic regression analysis of the clinical and epidemiological characteristics and risk factors was performed and the event-free survival Kaplan‑Meier curve was constructed.

This study was submitted to the Research Ethics Committee, with Certificate Presentation for Ethics Appreciation (CAAE: 20702313500005462).

## Results

A total of 892 patients were analyzed, characterized as asymptomatic or with atypical cardiac symptoms, who were submitted to MS from December 2005 to June 2011. Twenty-one patients were excluded due to inconclusive information in medical records or loss to follow-up during the study period, thus totaling 871 patients.

Of patients without known CAD (483 patients), 449 patients were completely asymptomatic since the consultation when MS was requested and 34 patients had symptoms considered atypical for CAD. Of the patients with known CAD (388 patients), 368 were totally asymptomatic since the consultation when MS was requested and 20 had atypical symptoms (p = 0.059).

After statistical analysis of the collected data, it was observed equivalence between the genders, with a prevalence of 454 (52.1%) male patients.

The Framingham score was applied to all assessed patients, with 611 (70.1%) being classified as having high cardiovascular risk, 190 (21.8%) as intermediate risk and 70 (8%) as low risk.

The prevalence of all analyzed variables with their respective percentages is shown in Table 1, depicting a large number of hypertensive (86.9%) and dyslipidemic (72.4%) patients.

**Table 1:** Prevalence of cardiovascular risk factors

Variables	Yes n (%)
Systemic arterial hypertension	757 (86.9)
Dyslipidemia	631 (72.4)
Smoking	106 (12.2)
OPAD/ carotid disease	58 (6.7)
Diabetes mellitus	293 (33.6)
CRF	37 (4.2)
iCVA	48 (5.5)
Previous CAD	388 (44.5)
LVEF < 50%	86 (9.9)
Family history of CAD	101 (11.6)

OPAD: occlusive peripheral arterial disease; CRF: chronic renal failure; iCVA: ischemic cerebrovascular accident; CAD: coronary artery disease; LVEF: left ventricular ejection fraction.

When evaluating the MS characteristics, it was observed that of the examinations performed, 385 (44.2%) were submitted to physical stress through exercise test and 486 (55.8%) were performed with pharmacological stimulation, through dipyridamole administration. At the ischemia-inducing tests for the MS, of the 871 tests analyzed, 189 (21.7%) showed abnormalities suggestive of ischemia due to the presence of ECG alterations and / or symptoms suggestive of ischemia.

The prevalence of normal MS, fixed low uptake suggestive of fibrosis and reversible low uptake suggestive of ischemia in the assessed patients was 511 individuals (58.6%), 227 (26.1%) and 133 (15.3%), respectively. Table 2 shows the prevalence of the results obtained by scintigraphy, including the ischemia-inducing test.

**Table 2:** Analysis of the results of altered ischemia-inducing tests and results of myocardial scintigraphy (MS)

Result	Yes n (%)
Altered ischemia-inducing test	189 (21.7)
Normal MS	511 (58.6)
Fixed low uptake	227 (26.1)
Reversible low uptake	133 (15.3)

Altered ischemia-inducing test: suggestive of ischemia.

Statistical analysis of the association between the clinical and epidemiological variables and the result of the MS was performed, which were described as normal, with fixed or reversible low uptake. The results are shown in Tables 3 to 5.

**Table 3:** Analysis on the association between clinical variables, risk factors, functional test results, events at the follow-up and normal myocardial scintigraphy (MS)

Variables	Normal MS n (%)	p value	OR	95%CI
Systemic arterial hypertension	463 (86.5)	0.757	0.919	0.611-1.381
Dyslipidemia	377 (70.5)	0.102	0.770	0.565-1.051
Smoking	61 (11.4)	0.396	0.832	0.551-1.256
OPAD/ carotid disease	35 (6.5)	0.889	0.953	0.553-1.642
Diabetes mellitus	173 (32.3)	0.338	0.860	0.649-1.147
CRF	16 (3)	0.250	0.462	0.238-0.899
iCVA	13 (2.4)	< 0.0001	0.214	0.112-0.411
Previous CAD	147 (27.5)	< 0.0001	0.149	0.110-0.202
LVEF < 50%	15 (2.8)	< 0.0001	0.108	0.061-0.192
Family history of CAD	74 (13.9)	0.009	1.841	1.158-2.927
Altered ischemia-inducing test	102 (19.1)	0.018	0.674	0.487-0.934
AMI	4 (0.7)	0.04	0.274	0.840-0.896
Death	1 (0.2)	0.034	0.124	0.014-1.066

OR: odds ratio; 95% CI: 95% confidence interval; OPAD: occlusive peripheral arterial disease; CRF: chronic renal failure; iCVA: ischemic cerebrovascular accident; CAD: coronary artery disease; LVEF: left ventricular ejection fraction; AMI: acute myocardial infarction.

**Table 5:** Analysis of the association between clinical variables, risk factors, functional test result, events in the follow-up and myocardial scintigraphy (MS) with reversible low uptake suggestive of ischemia

Variables	Ischemia MS n (%)	p value	OR	95%CI
Systemic arterial hypertension	122 (91,7)	0,093	1,799	0,938-3,450
Dyslipidemia	103 (77,4)	0,172	1,366	0,882-2,114
Smoking	16 (12)	1,000	0,985	0,558-1,736
OPAD/ carotid disease	7 (5,3)	0,574	0,748	0,332-1,686
Diabetes mellitus	55 (41,4)	0,046	1,481	1,015-2,162
CRF	7 (5,3)	0,487	1,311	0,564-3,050
iCVA	9 (6,8)	0,534	1,303	0,615-2,2753
Previous CAD	84 (63,2)	< 0,001	2,447	1,671-3,584
LVEF < 50%	18 (13,5)	0,153	1,542	0,885-2,689
Family history of CAD	13 (9,8)	0,557	0,799	0,432-1,476
Altered ischemia-inducing test	51 (38,3)	< 0,001	2,704	1,821-4,016
AMI	3 (2,3)	0,432	1,680	0,456-6,187
Death	1 (0,8)	1,000	1,111	0,124-9,582

OR: odds ratio; 95% CI: 95% confidence interval; OPAD: occlusive peripheral arterial disease; CRF: chronic renal failure; iCVA: ischemic cerebrovascular accident; CAD: coronary artery disease; LVEF: left ventricular ejection fraction; AMI: acute myocardial infarction.

Patients with CRF, ischemic CVA, previous known CAD and LVEF < 50% showed a greater association with MS suggestive of fibrosis, with statistical significance (Table 4).

**Table 4:** Analysis of association between clinical variables, risk factors, functional test result, events at the follow-up and myocardial scintigraphy (MS) with fixed low uptake suggestive of fibrosis

Variables	MS fibrosis n (%)	p value	OR	95%CI
Systemic arterial hypertension	194 (85.5)	0.492	0.846	0.547-1.309
Dyslipidemia	168 (74)	0.604	1.113	0.790-1.568
Smoking	30 (13.2)	0.557	1.138	0.724-1.790
OPAD/ carotid disease	20 (8.8)	0.162	1.541	0.877-2.208
Diabetes mellitus	75 (33)	0.870	0.964	0.699-1.329
CRF	17 (7.5)	0.007	2.526	1.299-4.912
iCVA	29 (12.8)	< 0.001	4.818	2.644-8.786
Previous CAD	181 (79.7)	< 0.001	8.307	5.776-11.947
LVEF < 50%	60 (26.4)	< 0.001	8.540	5.227-13.952
Family history of CAD	17 (7.5)	0.0290	0.539	0.312-0.929
Altered ischemia-inducing test	40 (17.6)	0.092	0.771	0.482-1.047
AMI	6 (2.6)	0.113	2.471	0.821-7.430
Death	4 (1.8)	0.043	5.758	1.047-31.651

OR: odds ratio; 95% CI: 95% confidence interval; OPAD: occlusive peripheral arterial disease; CRF: chronic renal failure; iCVA: ischemic cerebrovascular accident; CAD: coronary artery disease; LVEF: left ventricular ejection fraction; AMI: acute myocardial infarction.

When assessing the primary endpoint, patients with fixed low uptake had an increased chance of developing AMI during the analyzed period that was 2.4-fold higher than patients with normal MS, but without statistical significance. When evaluating the occurrence of death, of the six recorded deaths, four had MS with fixed low uptake, with a more than 5-fold higher chance of having this outcome, with statistical significance (odds ratio - OR: 5.958; 95% confidence interval - 95%CI: 1.047 to 31.651; p = 0.043) (Table 4).

In the group of patients that had MS with reversible low uptake suggestive of ischemia, the analysis of the variables showed that diabetes mellitus, previous known CAD and altered ischemia-inducing test (suggestive of ischemia) were associated with the development of reversible low uptake, which was statistically significant, with p < 0.05. The incidence of death and AMI was not statistically significant for this group of patients (Table 5).

In the independent analysis of variables, for the occurrence of death and AMI it was observed that CRF and LVEF < 50% were predictors for the occurrence of death was statistically significant, with p = 0.001 and 0.031, respectively. The presence of factors such as smoking and LVEF < 50% was an independent predictor for the occurrence of AMI, with p = 0.037 and 0.039, respectively.

**Figure 1:**
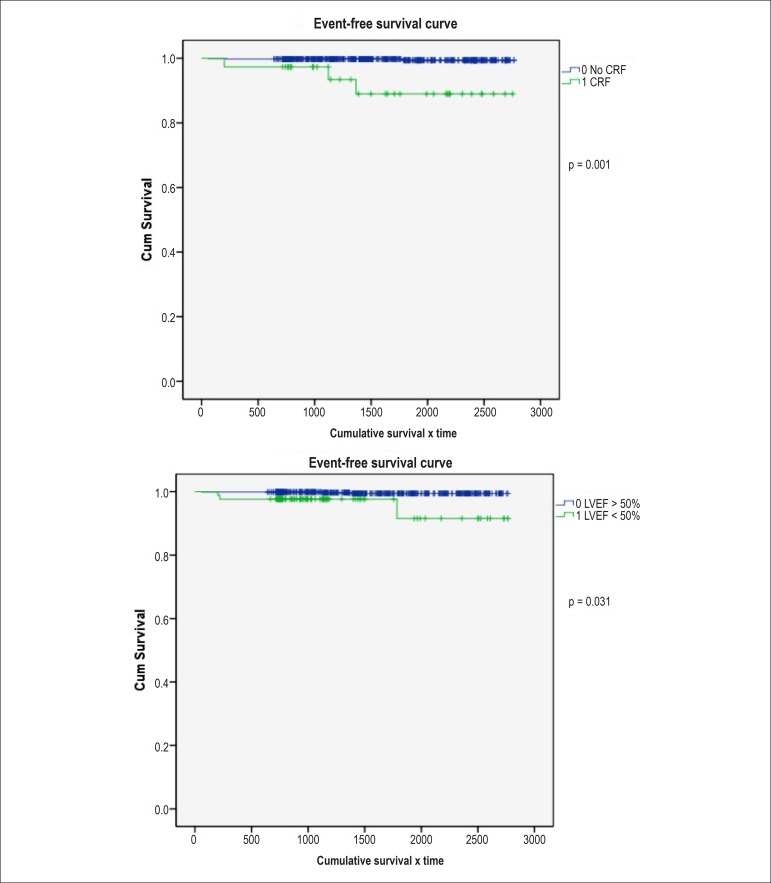
Kaplan-Meier curves for the occurrence of death vs. chronic renal failure (CRF) and vs. left ventricular ejection fraction (LVEF) < 50%

**Figure 2:**
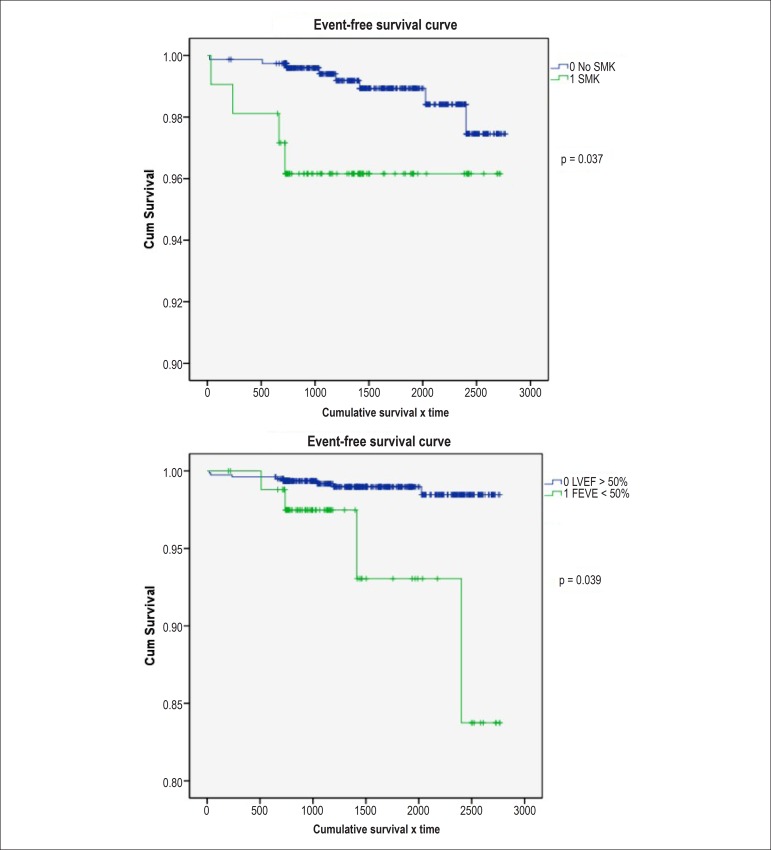
Kaplan-Meier curves for the occurrence of acute myocardial infarction (AMI) and vs. smoking (SMK) vs. left ventricular ejection fraction (LVEF) <50%

Figure 1 shows the Kaplan-Meier curve for the presence of CRF and LVEF < 50% for the outcome of death. Figure 2 shows the Kaplan-Meier curve for the presence of smoking and LVEF < 50% for the occurrence of AMI.

During the study period, 13 cases of AMI were obtained, of which four (30.8%) in the normal MS group, six (46.2%) in the MS group with fixed low uptake and three (23.1%) in MS group with reversible low uptake.

Six deaths were recorded, of which three were of cardiac origin. Of these three, two (33.3%) belonged to the fixed low uptake and one (16.7%) to the reversible low uptake group. The other three deaths were of noncardiac origin, with two (33.3%) in the fixed low uptake group and one (16.7%) in the normal scintigraphy group.

**Figure 3:**
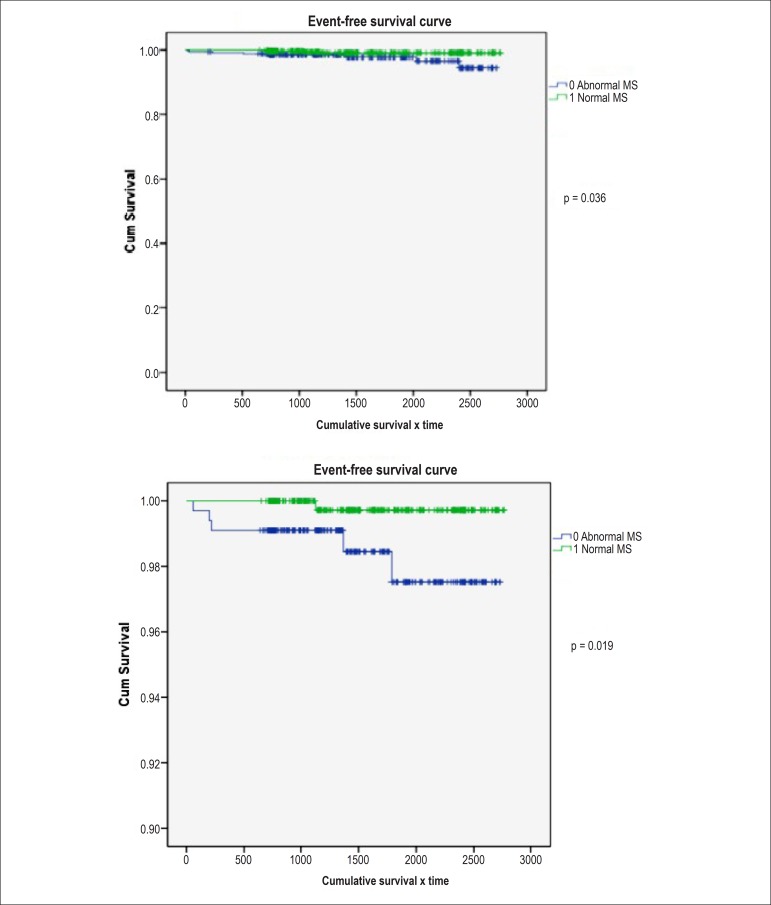
Event curves (acute myocardial infarction and death) in patients with normal or abnormal myocardial scintigraphy (MS)

During the mean follow-up of nearly 8 years, patients with normal MS showed better survival rates free of events such as death and AMI, when compared to that of patients with altered MS, with statistical significance, as demonstrated by the Kaplan-Meier curves (Figure 3).

## Discussion

Recent publications have sought to identify subgroups of asymptomatic patients that could benefit from the MS for the detection of ischemia, such as those with early CAD family history and patients with anginal equivalent, OPAD, erectile dysfunction, chronic renal failure and type 2 diabetes. However, the evidence for MS indication in these subgroups is still scarce^10^.

The American Society of Nuclear Cardiology (ASCN)^9^ recommends the performance of MS when screening for coronary disease in the following asymptomatic patient subgroups: those with high cardiovascular risk by the ATP III criteria; those with Agatston calcium score > 400; diabetics; patients with CRF; patients with incomplete myocardial revascularization; and as follow-up in patients with myocardial revascularization, 5 years after the surgery.

The results obtained in our study showed that MS should be performed in patients with high pretest probability, even without symptoms considered typical of ischemia, whether they have known coronary disease or not, as their outcome, when normal, is associated with low prevalence of major events in the follow-up.

The safety period after the performance of MS with normal result was well evaluated in a meta-analysis that included 17 studies with 8,008 patients, which showed that a normal MS result had a high negative predictive value for cardiac events for 3 years^14^.

A study published by Ottenhouf et al.^15^ evaluated 261 patients with known CAD and normal myocardial perfusion scintigraphy, who were followed for a mean period of 12 years, assessing as primary endpoint of death from all causes, cardiac death and AMI and / or cardiac death. They also analyzed independent predictors for the occurrence of events during this period. The results showed 94 (36%) deaths, 26 (10%) due to cardiac causes and 15 (6%) due to AMI; of the risk factors, age and diabetes were predictors for the event death from all causes, with statistical significance (OR: 1.05; 95% CI: 1.03 to 1.07; p < 0.05; and OR: 2.13; 95%CI: 1.23 to 3.71; p < 0.05, respectively).

Age and male gender were predictors for cardiac death, also with statistical significance (OR: 1.05; 95% CI: 1.01-1.10; p < 0.05; and OR: 2.45; 95% CI: 1.07 to 5.64; p < 0.05, respectively). Diabetes was associated with the occurrence of cardiac death and / or AMI, also with statistical significance (OR: 2.34; 95% CI: 1.03 to 5.30; p < 0.05). In the same study, the normal MS was associated with a lower probability of death from all causes, cardiac death and AMI and/or cardiac death, with annual event rates of 3.2, 0.9 and 1.2%, respectively.

Schinkel et al. ^16^, in another recent study, evaluated 233 patients with suspected or known CAD who underwent MS with^99m^ Tc-setamibi and had normal myocardial perfusion. They were followed for a mean of 15.5 years for the occurrence of death from all causes, cardiac death, AMI, and major cardiac events, defined as the occurrence of cardiac death, myocardial infarction and need for myocardial revascularization. Among the 233 patients, there were 41 (18%) deaths from all causes, of which 13 were cardiac deaths; 18 (8%) had AMI; and 47 (20%) required myocardial revascularization, of which 7% were submitted to cardiac surgery and 13% to PCI. The event annual rates for death from all causes, cardiac death and / or AMI and the presence of major cardiac events were 1.1, 0.3, 0.7 and 1.8%, respectively.

Factors such as age, male gender and diabetes were independent predictors for the occurrence of death from all causes, with statistical significance (OR: 1.06; 95% CI: 1.04-1.09; p < 0.001; OR; 2.70; 95% CI: 1.45 to 5.03; p = 0.002; and OR: 3.06; 95% CI, 1.22 to 7.65; p = 0.02, respectively). Factors such as male gender and diabetes were also independent predictors for the occurrence of major cardiac events with statistical significance (OR: 2.61; 95% CI: 1.11 to 6.14; p = 0.03; and OR: 6.93; 95% CI: 2.18 to 22.04, p = 0.01, respectively). It was concluded that patients with known or suspected CAD showed a favorable outcome during the analyzed period, especially during the first 5 years.

In comparison, this study showed normal MS in 511 patients (58.6%); 388 (44.5%) of them had known CAD, 293 (33.6%) patients had diabetes mellitus and 611 (70%) were classified as high risk by the Framingham score. Nevertheless, the group with normal MS showed lower probability of events such as death (p = 0.019) and AMI (p = 0.036) during the analyzed period with statistical significance, suggesting that patients with normal MS, even though they are at high cardiovascular risk, are less likely to have major events in the follow-up over a mean period up to 8 years.

In this study, when analyzing which variables were independent predictors of events such as death and AMI, it was verified that the presence of CRF and LVEF < 50% were risk factors associated with death, with statistical significance, with p = 0.001 and p = 0.031, respectively. For the occurrence of AMI, risk factors such as smoking and LVEF < 50% were associated with higher probability of this event, also with statistical significance, with p = 0.037 and 0.039, respectively.

It was also observed that patients with fixed low uptake in the MS showed higher probability of death outcome, with p = 0.043. Risk factors such as CRF, previous CVA, known CAD and LVEF < 50%, were, in turn, associated with the occurrence of fixed low uptake, suggestive of fibrosis.

### Study limitations

In this group of patients that was totally asymptomatic or without typical symptoms of ischemia since the medical consultation when the scintigraphy was requested, 44.5% had known coronary disease. In fact, the aim of this study was to evaluate events in patients without typical symptoms, and not in individuals without typical symptoms and without known CAD; therefore, the group with known CAD was not excluded. The idea for this study appeared when we discovered that, unlike what is recommended by the appropriate use criteria, many patients in our institution undergo scintigraphy even when they are asymptomatic and we tried to retrospectively determine whether the method discriminated events in asymptomatic patients, with most of them being considered as high risk according to the Framingham score. We observed that the method is of great value to discriminate major events in the follow-up.

Scintigraphy was analyzed qualitatively and magnitude (intensity and extent of perfusion findings) was not evaluated. The exercise test was interpreted as suggestive of ischemia due to the presence of suggestive clinical signs and symptoms and the presence of ECG alterations, whereas prognostic scores, such as the Duke score, was not analyzed.

## Conclusion

The results obtained suggest that in the group of patients without typical symptoms (asymptomatic or with atypical symptoms), submitted to myocardial scintigraphy, the occurrence of major cardiac events such as death and acute myocardial infarction was small. The event-free period after normal myocardial scintigraphy in this group was 7.5 years. The presence of perfusion alterations suggestive of fibrosis in the myocardial scintigraphy was associated with an increased number of deaths at follow-up and might, therefore, be considered an adjunctive risk factor for more detailed patient assessment and monitoring. The occurrence of acute nonfatal myocardial infarction showed no statistically significant difference between the groups of patients with normal perfusion, suggestive of fibrosis or ischemia.

Risk factors associated with perfusion abnormalities suggestive of fibrosis in the assessed group were chronic renal failure, ischemic stroke, known prior coronary artery disease and left ventricular ejection fraction < 50%. Diabetes, previous coronary artery disease and ischemic test (exercise or dipyridamole test) showed to be associated with myocardial scintigraphy suggestive of ischemia, but patients with this alteration in the myocardial scintigraphy showed no statistical significance for death or acute myocardial infarction, probably because they were submitted to therapeutic optimization and/or some type of therapeutic intervention.

Risk factors considered to be independent predictors of death were chronic renal failure and left ventricular ejection fraction < 50%. Smoking and left ventricular ejection fraction <50% were independent predictors of nonfatal acute myocardial infarction, regardless of the myocardial scintigraphy result.
